# Supplementary material for: Evaluating turnaround time to improve clients’ satisfaction in the tuberculosis reference laboratory in Douala
Source: PLoS One. 2025 Jun 13;20(6):e0323917. doi: 10.1371/journal.pone.0323917 (PMC12165355; doi:10.1371/journal.pone.0323917)
Supplement: Attachment — Submitted filename: AREBUT~2.DOC [file pone.0323917.s003.DOC]

A rebuttal letter for the manuscript titled: Evaluating Turnaround Time to Improve Clients' Satisfaction in the Tuberculosis Reference Laboratory in Douala submitted to PLOS ONE reference PONE-D-24-50358R1 - [EMID:551da529ab2a49a0]

| **SN** | **Reviewers’ preoccupation** | **Response provided** |
| --- | --- | --- |
| 1 | 1. We note that there is identifying data in the Supporting Information file <    TAT data.xlsx>. Due to the inclusion of these potentially identifying data, we have removed this file from your file inventory. Prior to sharing human research participant data, authors should consult with an ethics committee to ensure data are shared in accordance with participant consent and all applicable local laws. | Thanks, the data set has been reviewed, and all personal identifying data has been removed. |
|  |  |  |
|  |  |  |
|  |  |  |
